# The impact of second-generation androgen receptor pathway inhibitors on skeletal muscle morphology and strategies to mitigate their effects in prostate cancer patients

**DOI:** 10.3389/fresc.2025.1655422

**Published:** 2025-10-03

**Authors:** Sarah Edwards, Tea Lulic-Kuryllo, Anupam Batra

**Affiliations:** ^1^ Office of Research, Waterloo Regional Health Network, Kitchener, ON, Canada; ^2^ Department of Oncology, Waterloo Regional Health Network, Kitchener, ON, Canada

**Keywords:** metastatic prostate cancer, metastatic castrate sensitive, muscle morphology and function, muscle strength, physical function, exercise

## Abstract

The standard of care for metastatic castrate sensitive prostate cancer (mCSPC) involves the use of doublet therapies, which prolong survival and delay disease progression. Doublet therapies include the addition of second-generation androgen receptor pathway inhibitors (ARPIs) to androgen deprivation therapy (ADT). ADT monotherapy has been associated with adverse effects on skeletal muscle morphology, muscle strength, and physical function. Our findings suggest that the addition of ARPIs to ADT may further exacerbate these adverse effects. This review provides an overview of the current evidence to initiate exercise during treatment as an intervention to mitigate these adverse effects. Despite growing research in exercise oncology, research on the effects of exercise in men with mCSPC treated with doublet therapy is lacking. Much of the current supporting evidence is based on men with metastatic castrate resistant prostate cancer. Nonetheless, this review examines the available research on the efficacy and benefits of participating in a regimented exercise program in men with metastatic prostate cancer. We highlight the emerging evidence that exercising during treatment has the potential to protect against the adverse effects of doublet therapy. Future research to uncover the effects of different doublet therapies on muscle health in mCSPC is needed. Moreover, an improved understanding of the optimal training dose and timing that would elicit the most optimal benefits on muscle health in men with mCSPC is required.

## 1 Introduction

Prostate cancer is one of the most prevalent malignancies in men worldwide. It is estimated that new cases will rise from 1.4 million in 2020 to 2.4 million by 2040 (1). Approximately one-third of prostate cancers are diagnosed as metastatic. Historically, androgen deprivation therapy (ADT) was the cornerstone of systemic treatment for metastatic prostate cancer. ADT, or castration therapy, inhibits gonadotropin release by suppressing circulating testosterone levels to achieve chemical castration. However, the standard of care for metastatic prostate cancer has evolved to include androgen receptor pathway inhibitor (ARPI) therapies in combination with ADT (also known as doublet therapy).

Doublet therapies combine ADT with hormonal drugs, such as enzalutamide, abiraterone, apalutamide, or darolutamide. Most ARPIs (enzalutamide, apalutamide, and darolutamide) inhibit ligand binding at the level of the androgen receptor, further preventing the upregulation of genes that promote invasiveness and metastatic potential (2). This therapeutic intensification has demonstrated superior efficacy in prolonging survival and delaying disease progression compared to ADT alone (2). However, the concurrent interference of the cellular pathways at the level of the androgen receptor with the suppression of testosterone can evoke multiple adverse side effects contributing to the reductions in muscle mass and strength, and subsequent impairments in physical function (3, 4). While ADT alone is known to negatively affect muscle health and reduce patient quality of life (5), the intensification with ARPIs further inhibits androgen signaling, potentially exacerbating the toxic effects of ADT.

This review evaluates the impact of doublet therapies on muscle health in metastatic castrate sensitive prostate cancer (mCSPC) patients. We specifically focus on mCSPC patients because it has received relatively less research attention than metastatic castrate resistant prostate cancer (mCRPC) patients. While mCSPC implies a disease that is sensitive to androgen ablation, nearly all patients treated with ADT will ultimately develop castrate resistant disease (6), which is more aggressive and has a poor prognosis (7). Given that the first ARPI (apalutamide) was approved for use in mCSPC patients in 2019 (8), the impact of these agents in this population is a relatively understudied research area. Assessments of muscle health in mCSPC can provide powerful clinical insights into patient survival (9, 10) and treatment response (11). They may be particularly relevant as many mCSPC patients are at risk of or have already developed sarcopenia before initiating ARPI treatment, a condition that can predispose them to an increased risk of falls and fractures, hospitalization, increased frailty, and other adverse events (9).

This review also explores the current evidence regarding whether initiating an exercise program before or during doublet therapy can mitigate treatment-induced toxicities on the muscle. It is important to acknowledge that a patient's response to ARPIs and exercise interventions can be influenced by factors such as age, baseline fitness level, and comorbidities, which are out of the scope of this review. The insights gained from this review will assist in ongoing improvement to the standard of care therapy for men with mCSPC by identifying ways to counteract the declines in muscle health. Ultimately, this review provides a foundation for future research investigating the impact of incorporating a supervised exercise program into the standard of care for mCSPC patients during treatment.

## 2 Overview of skeletal muscle function in the context of androgen-receptor signaling

Androgens promote myogenic differentiation by inducing the transition of mesenchymal pluripotent cells to myogenic cells. Specifically, serum testosterone and other androgens bind to intracellular androgen receptors within muscle cells. Upon binding, the androgen receptor moves from the cytoplasm into the nucleus where it binds to hormone response elements that upregulate the transcription of genes that promote myogenesis (12). In healthy individuals, skeletal muscle mass is maintained through the achievement of homeostasis between the androgen receptor–β-catenin and insulin-like growth factor I–Akt–mammalian target of rapamycin [mTOR] pathways that promote muscle growth and the TGF-β–SMAD, ubiquitin–proteasome, and autophagy pathways that promote muscle atrophy (13).

ADT, when delivered alone, decreases circulating testosterone levels, reducing the potential for muscle hypertrophy by decreasing the resting rate of muscle protein synthesis (14). It is known that decreasing androgen levels reduces the activation of androgen receptors, leading to a reduction in mTORC1 signaling. mTORC1 is a central regulator of muscle protein synthesis and was identified as a major contributor to skeletal muscle hypertrophy in response to an increased workload (15–17). Therefore, a reduction in mTORC1 signaling with ADT contributes to a loss in muscle mass. However, the relationship between ADT disruption in these signaling pathways and muscle loss is complex and not well understood (18).

Previous research has shown that ARPIs act through different biological mechanisms. Abiraterone prevents androgen synthesis through the inhibition of cytochrome P-450c17 (CYP17), a critical enzyme in androgen biosynthesis (19). In contrast, enzalutamide and apalutamide inhibit the signaling pathway, specifically by blocking androgens from binding to androgen receptors (20, 21). Darolutamide acts through a similar mechanism to enzalutamide and apalutamide but has a tighter bond to the androgen receptor allowing for stronger suppression of androgen induced cell growth (22). Unlike other ARPIs, darolutamide minimally crosses the blood-brain barrier (23). Given these distinct therapeutic mechanisms, it is plausible that each ARPI, when combined with ADT, has a different effect on androgen and androgen receptors. Studies have shown that in men with mCRPC, treatment with abiraterone leads to significant and rapid reduction in circulating androgens (24) and testosterone (25). This rapid reduction appears to lead to more rapid muscle atrophy compared to ADT alone (4). Similarly, enzalutamide has been shown to result in a more rapid loss of skeletal muscle compared to ADT (4). Consequently, these mechanistic differences may lead to varying effects on skeletal muscle morphology, strength, and overall physical function.

## 3 Effects of ADT on muscle health

Men undergoing ADT often experience a significant loss in muscle mass and strength, which can lead to sarcopenia and subsequent declines in physical function. Loss of muscle mass and strength typically manifests within 3–6 months of initiating ADT treatment (26) and continues over the years (27). The adverse effects of ADT on muscle health are summarized in Table 1.

**Table 1:** A summary of published studies reporting changes in muscle morphology, strength, and physical function in men with prostate cancer receiving ADT only or doublet therapies.

Author	Sample size	Treatment	Assessment time	Outcome measure	Findings
Fischer et al. (28)	54 men with mCRPC	ADT + Enzalutamide: *N* = 37 ADT + Abiraterone: *N* = 17	Patients were already on ADT—Median time on ADT: 30 months *Baseline:* before ARPI initiation *Follow-up assessment:* Median time on enza/abi 10.8 months	Muscle morphology (Skeletal muscle loss; CT at L3 level)	ADT + ENZA: ↓5.2% compared to baseline ADT + ABI: ↓3.0% compared to baseline ≠ in skeletal muscle loss between two groups
Gonzalez et al. (29)	62 men with PCa on ADT (4 metastatic; 15 missing stage) 86 men with PCa not on ADT (no metastatic; 1 missing stage)	ADT monotherapy	*Baseline*: before ADT initiation or within 20 days of ADT initiation) *Follow-up assessment:* 6 and 12 months	Muscle strength (handgrip strength) Physical function (Chair Rise Test)	↓ 5.3% handgrip strength at 12 months in PCa on ADT (compared to baseline) ↓ Chair Rise Test at 6 (25%) and 12 months (29.5%) in PCa on ADT compared to controls ≠ Chair Rise in PCa on ADT at 6 and 12 months compared to baseline
Houben et al. (30)	Exercise + placebo: 30 PCa patients (14 with bone metastases) Exercise + protein supplementation: 30 PCa patients (11 with bone metastases) PCa control: 36 (19 with bone metastases)	ADT monotherapy	Control group assessed at: *Baseline*: 31 ± 6 days on ADT *Follow-up assessment:* 20 weeks post-intervention	Muscle morphology (Quadriceps muscle CSA (CT) Muscle strength (1 RM leg press and leg extension) Physical function (TUG,30-Second Chair Stand Test, Stair Climb test)	Control ONLY: ↓ 1.2 ± 2.5 cm^2^ of quadriceps muscle CSA ↓ in 1 RM leg press (5 ± 11%) and leg extension (6 ± 15%) ≠ in TUG, 30-Second Chair Stand Test, Stair Climb test
Lawen et al. (31)	55 men with metastatic prostate cancer	ADT alone: *N* = 16 ADT + abiraterone: *N* = 29 ADT + enzalutamide: *N* = 10	*Baseline:* before treatment initiation *Follow-up assessment:* median follow-up of 335 days post-hormone initiation	Muscle morphology (Skeletal muscle index (CT at L3 level)	ADT only SMI: ↓4.05 cm^2^/m^2^ ADT + Abiraterone SMI: ↓2.47 cm^2^/m^2^ ADT + Enzalutamide SMI: ↓3.13 cm^2^/m^2^
Overkamp et al. (32)	21 PCa patients 11-ADT (6 with bone metastases) 10-ADT + exercise (5 with bone metastases)	ADT monotherapy	Control group assessed at: *Baseline:* a mean of 39 ± 31 days post-ADT initiation *Follow-up assessment:* 20 weeks post-intervention	Muscle morphology (Type I and II muscle fiber size in vastus lateralis muscle (muscle biopsy), CSA of the quadriceps muscle (Leg CT)	CSA of Type I muscle fibers: ↓ from 7,401 ± 1,183 at baseline to 6,489 ± 1,293 μm^2^ at 20-week follow-up CSA of Type II muscle fibers: ↓6,225 ± 1,503 at baseline to 5,014 ± 714 μm^2^ at 20-week follow up ↓ ∼4.4% Quadriceps CSA compared to baseline
Owen et al. (33)	70 patients on ADT (45 advanced) 52 patients not on ADT (6 advanced) 70 age-matched healthy controls	ADT monotherapy	Mean time on ADT at assessment: 25 ± 36 months *Group comparison*	Muscle morphology (Muscle CSA of wrist flexors and extensors and gastrocnemius muscle (peripheral quantitative CT) Muscle strength (handgrip strength; chest press, leg press, seated row)	Wrist flexor/extensor muscle CSA: 5.2%–6% ↓ in ADT group compared to PCa controls Gastrocnemius muscle CSA: 3%–3.7% ↓ compared to PCa controls Hand grip strength: 15%–17% ↓ compared to both controls. Chest strength 15% ↓ in ADT group compared to both controls. Back strength 15%–16% ↓ in ADT group compared to both controls Leg muscle strength 15% ↓ in ADT group compared to healthy controls, but not PCa controls
Papadopoulos et al. (34)	110 men with mCRPC	ADT + ARPI (abiraterone (16.4%) or enzalutamide (40.9%) or chemotherapy	Participants were already on ADT for a mean of 6.2 ± 4.9 years *Assessment of sarcopenia: before initiating ARPI* CT taken 6 months before ARPI initiation (baseline)	Muscle morphology (SMI (CT) Muscle strength (handgrip strength) Physical function (gait speed via 4 meter walk)	↓ hand grip strength in 67.3% of participants ↓ gait speed in 32.7% of participants ↓ SMI in 82.7% of participants ↓ skeletal muscle density in 78.2% of participants
Pezaro et al. (35)	55 patients with mCRPC	ADT + abiraterone	*Baseline:* before abiraterone initiation *Follow-up assessment:* 7.5 months post-abiraterone initiation	Muscle morphology (Muscle cross-sectional area (CT at L3 level)	↓ 2.8%–4.3% skeletal muscle CSA (BMI > 30: 4.3% loss; BMI < 25: 2.9% loss; BMI 25–30: 2.8% loss)
Streckova et al. (36)	43 patient with metastatic prostate cancer (*N* = 14 metastatic hormone-sensitive prostate cancer; *N* = 29 mCRC)	ADT + abiraterone	*Baseline:* at initiation of abiraterone *Follow-up assessment:* Unspecified later time point	Muscle morphology (SMI (CT at L3 level)	↓ SMI at baseline (% change not reported) ↓ SMI at follow-up (% change not reported)

ADT, Androgen Deprivation Therapy; TUG, timed up and go; CSA, cross sectional area; 1 RM, one repetition maximum; CT, computed tomography; SMI, Skeletal Muscle Index; ENZA, Enzalutamide; ABI, abiraterone.

There is evidence that ADT leads to muscle atrophy in metastatic cancer patients. For example, Overkamp et al. (32) assessed the impact of ADT on vastus lateralis muscle fibers, revealing a 12% and 17% decrease in vastus lateralis Type I and Type II muscle fiber cross-sectional area (CSA), respectively, after only 20 weeks of ADT treatment. Furthermore, studies have shown significant declines in muscle CSA in quadriceps (2% to 4.4%), wrist flexor and extensor (5.2%–6.0%), and gastrocnemius (3.0%–3.7%). ADT has also been shown to reduce skeletal muscle index (SMI) (9, 31) and skeletal muscle density (9).

Beyond its effects on muscle morphology, ADT also negatively affects muscle strength and potentially physical function in men with metastatic prostate cancer. Previous findings have shown a reduction in handgrip strength (5.3% to 17%), leg press strength (5% to 15%), leg extension strength (6%), chest press strength (15%) and back strength (16%) (9, 29, 30, 33). The impact of ADT on physical function, on the other hand, is highly variable. Some studies have shown minimal declines in the chair rise test (0.6%–1.1%) (29), while others have shown no changes in Timed Up and Go and 30 s chair stand test (30). The variable effects of ADT on physical function may be attributed to differences in the duration of ADT, with declines in physical function more likely to be observed with longer periods of treatment. Collectively, these findings demonstrate a potential decline in muscle strength, which may be coupled with physical function impairments, underscoring the importance of monitoring and addressing muscle function in men with metastatic prostate cancer undergoing ADT.

## 4 Effects of doublet therapy (ADT and ARPI) on muscle health

Considering the adverse effects of ADT discussed previously, a logical extension is to hypothesize that the addition of ARPIs to ADT treatment would result in greater detriment to muscle morphology and subsequent declines in muscle strength and physical function. A few studies have investigated the effect of doublet therapies on these outcome measures, with most studies focusing on mCRPC (Table 1).

A limited number of studies have investigated the impact of doublet therapies on muscle morphology in metastatic prostate cancer patients. Lawen et al. (31) compared the effects of ADT alone to ADT + abiraterone or ADT + enzalutamide on SMI. In their study, SMI was quantified by computed tomography (CT) scan at the level of the L3 vertebrae at baseline and 6 months post-ARPI initiation. Although the authors did not specify the type of metastatic prostate cancer, they found that SMI decreased by 5%–7% across all groups after 6 months, irrespective of the treatment type. Similarly, Streckova et al. (36) investigated the effects of ADT and abiraterone on skeletal muscle mass quantified by CT scans at the level of the L3 vertebrae at the time of initiating abiraterone and at an unspecified time point during the study. The patient sample consisted of 29 patients with mCRPC and 14 patients with mCSPC. SMI was quantified from the combined estimate of the total cross-sectional area of the muscles at the L3 level. The authors reported a reduction in SMI at the time of abiraterone initiation and a further decrease in SMI at the unspecified later time point. Collectively, these limited findings suggest similar suppressive effects of treatments on SMI in a mixed group of metastatic prostate cancer patients.

Most of the available research has focused on investigating muscle health in patients with mCRPC, who have a poorer prognosis than mCSPC patients. Fischer et al. (28) investigated skeletal muscle mass in men who were receiving ADT and enzalutamide or ADT and abiraterone and prednisolone with a median ADT duration of 30 months. The authors reported a 5.2% decrease in skeletal muscle mass in men receiving ADT and enzalutamide and a 3.0% loss in those receiving ADT and abiraterone and prednisolone at a median of 10.8 months post-initiating ARPIs. The authors concluded that, irrespective of the hormonal drug used in doublet therapy, the effects on the muscle mass were equivalent. Similarly, Pezaro et al. (35) investigated how abiraterone affects muscle CSA at the level of the L3 vertebrae in men with mCRPC. They showed significant skeletal muscle loss (∼2.9% in BMI < 25 or 25–30; ∼4.3% in BMI > 30) after about 7.5 months of abiraterone use in mCRPC patients with the greatest loss observed in those with a BMI greater than 30. Collectively, these limited findings suggest that the addition of ARPIs impacts skeletal muscle morphology in metastatic prostate cancer patients, and the effects on muscle mass appear to be similar across different medications. However, more research is needed to elucidate the effects of different hormonal medications on muscle strength and physical function.

## 5 Initiating exercise to maintain muscle health

The benefits of exercise interventions in prostate cancer patients have been shown in several studies. Engaging in resistance training during ADT significantly improves muscle morphology (37–39), decreases prevalence of sarcopenia (37), improves physical function (30, 38, 39), and increases muscle strength (34, 37–40). Collectively, these studies support the inclusion of a supervised exercise program in the standard of care to counteract the negative effects of ADT on muscle health.

Most studies investigating the effects of exercise in men with metastatic prostate cancer have focused on safety, feasibility, and efficacy, showing that exercise is feasible and safe in this population, including those with bone metastases (41–46). While no studies have investigated the effects of exercise on muscle health in men with mCSPC, the knowledge gained from studies in men with mCRPC can be translated to this group. Therefore, this section will summarize the available literature (Table 2) pertaining to the effects of exercise on muscle health in metastatic prostate cancer patients without a specific focus on men with mCSPC.

**Table 2:** A summary of published studies investigating exercise as an intervention to mitigate adverse effects on muscle function in men with prostate cancer receiving ADT or doublet therapy.

Author	Type of prostate cancer and sample size	Treatment	Intervention	Assessment timeline	Measure	Primary outcome
Brown et al., (41)	24 men with mCRPC	ADT + abiraterone ADT + enzalutamide	12 week home based exercise program	Baseline 12-weeks post-intervention	Physical function (6 MWT, 30 s Sit to Stand)	↑ 6 MWT distance by 13.3% ↑ 30 s sit to stand by 25%
Evans et al. (42)	41 men with unspecified metastatic prostate cancer 20 men in exercise intervention 20 men in wait-list control	93% of participants on ADT monotherapy	Use of the ExerciseGuide website-individualized multimodal exercise prescription via text and videos for 8 weeks	Baseline Follow-Up: 9 weeks	Physical function (400 m walk test, TUG, 5 time sit to stand) Muscle strength (1 RM leg extension and chest press)	After exercise intervention- 400 m walk test: ↓0.8 min Chest press 1 RM: ↑8.5 kg ≠between control and exercise group in 1 RM leg extension, TUG, and 5 times sit to stand
Galvao et al. (43)	50 men with non-bone metastatic prostate cancer Acute ADT: *N* = 16 Chronic ADT: *N* = 34	ADT monotherapy	12 week progressive resistance and aerobic exercise (immediate or delayed)	Group comparisons	Muscle morphology (Appendicular skeletal mass (DXA) Muscle strength (chest and leg press, leg extension and seated row) Muscle endurance 70% 1 RM (chest press and leg press) Physical function (400 meter walk, 5 time sit to stand, 6 meter usual and fast walk)	Acute ADT: ASM: 0.5 kg ↑ Chronic ADT: ASM: 0.6 Kg ↑ Both groups: 400 m walk: ↓ (values not reported) 1 RM chest press strength ↑ in chronic ADT group (values not reported) ≠ between groups in 1 RM leg press, 5 time sit to stand, and 6-meter usual and fast walk
Hanson et al. (47)	22 men with mCRPC	Mixed sample: ADT + abiraterone (6/16 patients) ADT + Enzalutamide	12 week home-based intervention (progressive walking and resistance training)	Before and after intervention	Muscle morphology (Vastus lateralis CSA and muscle quality (ultrasound) Muscle strength (1 RM chest press and 1 RM leg press) Physical function (TUG, 6 meter and 400 meter walking test, Stair climb)	Vastus lateralis CSA: ↑ 22% ≠ in muscle quality ≠ in 1 RM chest press, TUG, 6 meter walk, 400 meter walk, stair climb
Houben et al. (30)	Exe + pla: 30 PCa patients (14 with bone metastases) Exe + pro: 30 PCa patients (11 with bone metastases) PCa control: 36 (19 with bone metastases)	ADT monotherapy	Supervised progressive whole-body resistance exercise training (20 week duration)	Baseline (31 ± 6 days on ADT) 20 weeks post	Muscle morphology (Quadriceps muscle CSA (CT) Muscle strength (1 RM leg press and leg extension) Physical function (TUG, 30-Second Chair Stand Test)	Exercise + Placebo: 1 RM leg press: ↑ 12% ± 14% 1 RM leg extension: ↑ 19% ± 15% Quadriceps CSA: ↑2.0 ± 3.0 cm^2^ Exercise + Protein: 1 RM leg press: ↑ 13% ± 11% 1 RM leg extension: ↑ 15% ± 19% Quadriceps CSA: ↑1.9 ± 2.7 cm^2^ Control: 1 RM leg press: ↓ 5% ± 11% 1 RM leg extension: ↓ 6% ± 15% Quadriceps CSA: ↓1.2 ± 2.5 cm^2^
Kenfield et al., (46)	25 men with mCRPC *N* = 9 aerobic exercise *N* = 8 resistance exercise *N* = 10 standard of care (no exercise)	Past or current treatment with doublet therapy (ADT + enzalutamide or ADT + abiraterone)	12 week 20–30 min of moderate to vigorous intensity exercise 3–5 days/week Patients randomized into HIIT, resistance training or standard of care	Baseline 12-weeks post-intervention	Muscle strength (1 RM chest press; 1 RM leg extension; 1 RM seated row) Physical function (400 meter walk test and steep ramp test on bike)	Resistance training group (to control): 1RM chest press: ↑ 13.8 lbs median difference 1 RM leg press: ↑ 95 lbs median difference 1 RM seated row: ↑ 15 lbs median difference ≠ in stair climb test, steep ramp test, 400 m walk test, repeated sit to stand Aerobic group (to control): 400 m walk test: ↓ 5 s median difference Steep ramp test: ↑ 10 watts median difference ≠ group differences in 1 RM chest press, 1 RM leg extension, 1 RM seated row, stair climb test, repeated sit to stand
Overkamp et al. (32)	21 PCa patients 11-ADT (6 with bone metastases) 10-ADT + exercise (5 with bone metastases)	ADT monotherapy	20-week, twice-weekly, progressive whole-body resistance exercise training program	Baseline 20 weeks post-intervention	Muscle morphology (Type I and II muscle fiber size in vastus lateralis muscle (muscle biopsy), CSA of the quadriceps muscle (Leg CT)	Quadriceps muscle CSA: ↑ ∼4.7% in exercise group Type I muscle fiber CSA: ↑ from 6,700 ± 1,464 to 7,772 ± 1,319 μm post-exercise Type II muscle fiber CSA:↑ from 5,248 ± 892 to 6,302 ± 1,385 μm^2^ post-exercise

ADT, Androgen Deprivation Therapy; TUG, timed up and go; 6MWT, 6 min walk test; CSA, cross sectional area;1RM, one repetition maximum; DXA, dual-energy x-ray absorptiometry; CT, computed tomography; SMI, Skeletal Muscle Index; ASM, Appendicular Skeletal Mass; HITT, high intensity interval training.

Several studies in men with metastatic prostate cancer on ADT monotherapy have shown positive effects of exercise on muscle health (Table 2). Exercise training has been shown to increase Type I and Type II muscle fiber CSA in the vastus lateralis by 16%–20% (32), and quadriceps CSA by approximately 5% (30, 32). Studies have also shown an increase in appendicular skeletal muscle mass by 0.5 kg to 0.6 kg (48), as well as muscle strength by 12% to 19% (30, 42, 48).

In contrast, the effects of exercise on physical function in men on ADT monotherapy are more variable. While some tests, particularly more endurance focused ones such as the 400-meter walk test, have shown improvements post-exercise (13–48 s time reduction) (42, 48), other tests, such as the Timed Up and Go and a 30 s stair stand test, have shown little to no change following exercise (30, 48). This discrepancy may be because the physical function tests are not sensitive enough to detect subtle changes, or may capture multiple domains including physical function, muscle strength, balance and coordination. Nonetheless, these preliminary findings suggest that improvements in muscle morphology and muscle strength might not always translate into improvements in physical function.

Other research has investigated the effects of exercise on muscle health in metastatic prostate cancer patients on doublet therapy (Table 2). The CHAMP study by Kenfield et al. (46) randomly assigned 25 men with mCRPC to either supervised remote high-intensity interval training (aerobic exercise; *N* = 9), supervised remote resistance training (*N* = 8), or a standard of care control group (*N* = 10) for 12 weeks. The treatments received by the participants in this study were heterogeneous, with 56% of patients on ADT + abiraterone, 28% on ADT + enzalutamide, and other antiandrogen therapies. Physical function (stair climb, 400-meter walk, and repeated sit-to-stand) and strength (chest-press, leg press or extension, and seated row) were measured at baseline and 12 weeks. The resistance exercise group had greater improvements in 1RM chest-press (13.8 lbs median difference), leg press (95 lbs median difference), and seated row (15 lbs median difference) compared to the control group. In contrast, the aerobic exercise group had greater improvements in steep ramp test (10 watts median difference) and 400-m walk times (5 s median difference) compared to the control group. These findings suggest divergent effects of different exercise modalities on strength and physical function. Similarly, Brown et al. (41) investigated the effect of a 12-week home-based exercise program in men with mCRPC receiving ADT + abiraterone acetate or ADT + enzalutamide. The exercise program included both resistance and aerobic components at moderate intensity. The authors assessed physical function using the 6-min walk test and the timed sit-to-stand test. They demonstrated that physical function improved significantly, with a 13.3% increase in 6-min walking distance and a 25% improvement in the timed sit-to-stand test.

However, other studies have reported variable results. For example, Hanson et al. (47) investigated the effects of a 12-week home-based exercise program in men with mCRPC on ADT + enzalutamide or ADT + abiraterone. The exercise program included progressive walking and resistance training. The authors observed a 22% increase in vastus lateralis CSA but no changes in muscle strength or physical function following exercise. Collectively, the findings from this small sample of studies suggest that home-based exercise programs can be effective in improving muscle morphology and preventing declines in physical function and muscle strength in men with mCRPC on doublet therapy. However, more studies are needed to confirm these findings.

## 6 Quality of life

While overall survival for men with metastatic prostate cancer continues to improve, optimizing health-related quality of life (HRQoL) for patients during and after treatment has become increasingly relevant. At least two studies assessed HRQoL in men with mCSPC receiving ADT + enzalutamide (49) or ADT + apalutamide (8). Based on the Functional Assessment of Cancer Therapy-General and Prostate-specific questionnaire (FACT-G and FACT-P) and the European Organization for Research Treatment of Cancer Quality of Life Questionnaires (EORTC QoL-C30), HRQoL was preserved in patients treated with doublet therapy (8, 49). This preservation in HRQoL has also been reported in mCRPC studies using the same measures (50, 51). Ronningas et al. (51) observed a significant association between a high physical symptom burden and HRQoL, reporting a correlation between low quality of life and high number of physical symptoms. Thus, investigating strategies aimed at mitigating these physical adverse effects, such as exercise, may be beneficial in enhancing HRQoL and patient care.

A limited number of studies have investigated the impact of exercise on patient-reported HRQoL in patients with mCRPC. One study by Langlais et al. (52) examined the impact of a remotely monitored 12-week exercise program on HRQoL in men with mCRPC. The authors observed minimal changes in HRQoL, regardless of participation in an exercise program. These findings suggest that the duration or intensity of the exercise intervention may not have been sufficient to elicit an impact on HRQoL.

In contrast, other studies have shown more promising results. Dawson et al. (37) investigated changes in HRQoL following a 12-week exercise intervention in a mixed population of both early-stage and metastatic prostate cancer. Specifically, the exercise group had a 12.5% increase in general quality of life, while the control group had a 3% decrease. Similarly, prostate specific quality of life increased by 12.3% in the exercise group and decreased by 2.8% in the control group. Brown et al. (41) also reported improvements in patient-reported HRQoL in mCRPC patients who participated in a 12-week home-based exercise program. Collectively, these findings support the potential role of exercise in enhancing quality of life among a more advanced prostate cancer population. However, further research is needed to investigate the specific benefits of exercise in optimizing HRQoL in mCSPC patients.

## 7 Limitations of previous research

There are several limitations to the studies described in this review. The most significant limitation is the lack of rigorous and consistent assessment of sarcopenia. Most studies have only evaluated the effects of treatment or exercise on muscle CSA at the L3 vertebrae level without concomitant evaluations of muscle strength or physical function. However, the definition of sarcopenia was updated in 2010 to include a measure of muscle function in addition to muscle mass (53). The exclusion of functional muscle tests (e.g., muscle strength or physical function) can significantly alter the proportion of patients classified as sarcopenic (9). For example, Papadopoulos et al. (9) showed that using only muscle mass measurements in mCRPC resulted in a sarcopenia diagnosis in 82.7% of patients in their study. However, the number of patients diagnosed with sarcopenia significantly decreased to 27.3% when the muscle function tests were considered. Future research should also include muscle quality assessment, which is a part of the sarcopenia definition and influences muscle function.

Another significant limitation is the lack of information on the type of metastatic prostate cancer. Following the TITAN trial in 2019, apalutamide was the first ARPI to be FDA approved for the treatment of mCSPC (8) and as a result, very few studies have investigated the effects of ARPI treatment in this population. Distinguishing between mCSPC and mCRPC is important, as these populations have different prognoses, treatment durations, and exposure to various ARPIs. Therefore, the effects on muscle health may be different between these two populations. Furthermore, a lack of detailed patient characteristics, including ARPI type, comorbidities, age, and baseline physical activity levels, limits the ability to interpret findings and implement them in a clinic.

Lastly, future research in metastatic prostate cancer aiming to investigate the impact of treatment on muscle health should consider the addition of baseline assessments prior to or within the first few weeks of treatment initiation. This would allow for better insights into the longitudinal impact of different hormonal agents on muscle characteristics. Researchers should consider adopting a pragmatic longitudinal approach in order to preserve real-world feasibility as well as be able to capture the true effects of ADT prior to ARPI initiation to determine the veritable consequences of additive therapy with ARPIs on muscle morphology and physical function.

## 8 Clinical takeaways

Findings from this review suggest that rigorous evaluation of muscle health in men with metastatic prostate cancer may help to detect subclinical sarcopenia. The clinical harms induced by a sarcopenic phenotype have been well-established and include an increased risk of emergency department utilization (9), hospitalization (54), falls and fractures (55), frailty (56), accelerated rate of disease progression (9, 57, 58), and at least grade 3 (severe) treatment-related toxicity (9). Sarcopenia was also associated with decreased overall survival (9, 11). Therefore, the assessment of sarcopenia at baseline and during hormonal therapy can help identify patients at high risk for detrimental effects and poor outcomes. If a patient has sarcopenia, clinicians should consider referring them to an exercise program and other support services to improve their quality of life. Our review also highlights the significant potential of a well-designed exercise regimen to mitigate potential adverse effects associated with ADT monotherapy or doublet therapy. When introduced early in the treatment pathway, exercise may prevent a decline in muscle health, thereby counteracting the observed adverse effects of such agents.

Despite these promising findings, several areas require further research. Most studies to date have failed to comment on the underlying mechanisms contributing to changes in muscle health. Additionally, no studies have compared the effects of different ARPIs on muscle health, specifically in men with mCSPC. Moreover, the potential mitigation of adverse effects through exercise training in mCSPC patients remains understudied. Additional work is needed to identify optimal timing of exercise programs and how best to implement lifestyle modification for patients receiving doublet therapy. We hypothesize that early exercise intervention during the castration sensitive stage of the disease may have beneficial effects on musculoskeletal function relative to usual care.

Lastly, the majority of research to date has examined short-term musculoskeletal consequences in response to an exercise program that lasts 12–20 weeks. This brief intervention, followed by a short-term observation period, limits one's ability to draw conclusions regarding any sustainability in improvement or further decline in physical function beyond study timelines. Further research across these domains could help better understand longer-term consequences on muscle compartments and subsequently lead to the development of strategies to ensure lasting muscle and physical function. Finally, improved understanding of training dose, including optimal mode, duration and intensity of exercise, may ultimately translate to favourable muscle and physical function outcomes for men with metastatic prostate cancer.

## 9 Conclusions

This review examined emerging evidence supporting participating in a progressive resistance exercise program as a means to mitigate the adverse effects associated with doublet therapy. Evidence suggests that engaging in a progressive resistance training exercise program is not only safe in metastatic prostate cancer populations but serves to improve muscle morphology, strength and physical function throughout the course of treatment which has the potential to improve patient health-related quality of life regardless of which form of ARPIs is administered. Early initiation of an exercise program during the castration sensitive stage of disease may elicit beneficial effects or lessen the harm to musculoskeletal morphology and function. Further research should aim to establish the ideal training dose and sustainability after completion of the initial regimented exercise program.
